# Heparan Sulfate Proteoglycans in Immunity

**DOI:** 10.1002/pgr2.70048

**Published:** 2026-06-21

**Authors:** Moe Iwai, Pyong Woo Park

**Affiliations:** 1 Department of Medicine, Boston Children’s Hospital, Boston, Massachusetts, USA; 2 Department of Pediatrics, Harvard Medical School, Boston, Massachusetts, USA

**Keywords:** adaptive immunity, glypicans, heparan sulfate, infectious disease, inflammatory disease, innate immunity, proteoglycan, serglycin, syndecans

## Abstract

In immune cells, heparin was first identified in mast cells in the 1940s, followed by the discovery of heparan sulfate in lymphocytes and granulocytes during the 1970s and 1980s. Subsequent decades of research have revealed how heparan sulfate and heparin, conjugated to core proteins as heparan sulfate proteoglycans (HSPGs), act as versatile modulators of immune function. It is now known that HSPGs are expressed by virtually all leukocytes and bind hundreds of immune mediators, including cytokines, proteases, growth factors, antimicrobial peptides, and extracellular matrix (ECM) components. HSPGs on leukocyte surfaces and within intracellular granules, as well as those on non-immune cells and within the ECM, shape immune cell development and responses to both endogenous and exogenous threats. Because immune activity must be tightly controlled to prevent collateral tissue damage, HSPGs play critical modulatory roles—facilitating, competing, protecting, presenting, or allosterically regulating immune factors. Consequently, mutations in HSPG core proteins and heparan sulfate biosynthetic enzymes, or dysregulation of HSPG-mediated immune interactions, contribute to diverse pathologies, including primary immunodeficiencies, autoimmune disease, infection, inflammatory tissue injury, allergy, and cancer. This review will highlight key mechanisms through which HSPGs regulate the development and function of major immune cell types.

## 1 | Introduction

**FIGURE 1 |:**
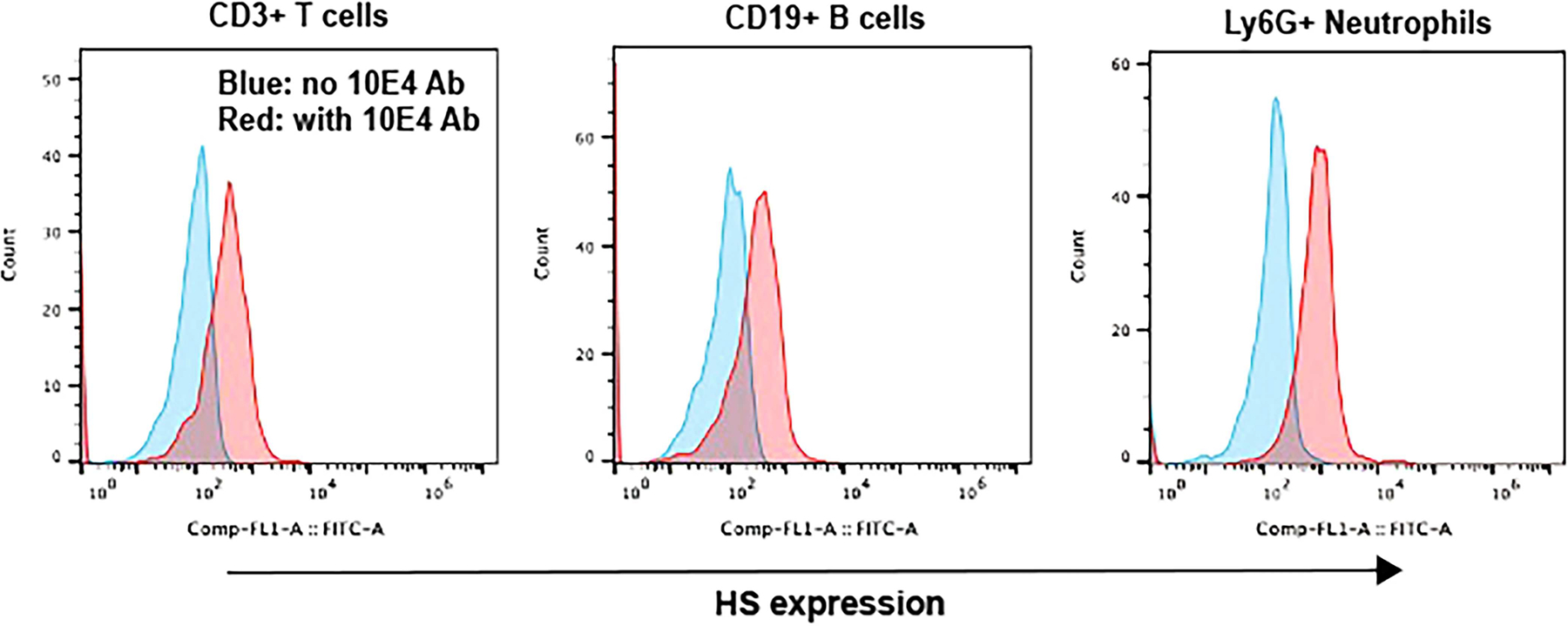
Expression of HS on immune cells. Mouse blood was collected by cardiac puncture, rapidly diluted in red blood cell (RBC) lysis buffer, and incubated for 5 min at room temperature. The remaining leukocytes were pelleted and stained for 30 min with 1 μg/mL Alexa 488-conjugated 10E4 anti-HS antibodies and 5 μg/mL of APC-conjugated anti-mouse CD3 (T cells), CD19 (B cells), or Ly6G (neutrophils) antibodies (Biolegend) or stained only with secondary leukocyte-specific antibodies for 30 min at room temperature. Surface expression of HS on each leukocyte subset was then assessed by flow cytometry.

Heparin (HP), initially referred to as “cephalin,” was identified in 1916 as a potent anticoagulant isolated from liver tissue. In immune cells, HP was first identified in mast cells in the 1940s [1, 2]. Heparan sulfate (HS) was first considered a contaminant of HP preparations but was later distinguished by its lower degree of sulfation and greater structural heterogeneity [3–5]. Flow cytometry analyses with the 10E4 anti-HS antibody demonstrate that circulating, mature immune cells express HS on their cell surface (Figure 1). All HS and HP chains are covalently attached to specific core proteins and exist as heparan sulfate proteoglycans (HSPGs) in vivo. Nearly all mammalian cells express one or more types of HSPGs, of which at least 17 have been identified. The 4 type I transmembrane syndecans (SDCs) and 6 glycosylphosphatidylinositol (GPI)-anchored glypicans (GPCs) make up the major families of cell surface HSPGs [6–9], while perlecan, agrin, and collagen XVIII are the main secreted HSPGs [10–12]. Serglycin is the main intracellular HSPG that carries HP chains [6], and the type I transmembrane proteins CD44 and betaglycan are part-time HSPGs that bear HS chains in certain contexts. HS consists of linear, unbranched disaccharide repeats of a uronic acid (glucuronic acid or iduronic acid) alternating with glucosamine residues that may be unsubstituted, *N*-acetylated, or *N*-sulfated [7, 8]. During HSPG biosynthesis, an unmodified HS/HP precursor is polymerized on specific Ser residues within the HSPG core protein, and then extensively and variably modified in the Golgi by *N*-deacetylation, *N*-sulfation, epimerization, and several *O*-sulfation reactions [7, 8], generating highly heterogeneous, mature HS chains and highly sulfated mature HP chains.

HSPGs bind and regulate many key factors implicated in immunity, including cytokines, antimicrobial peptides, proteases, growth factors, and ECM components [9–11]. They are expressed in a temporally and spatially regulated manner across diverse cell types and tissues, partly explaining their context-specific functions in vivo. HSPGs interact specifically and noncovalently with proteins mainly through their HS chains, and in some cases through their core proteins, thereby modulating the activity, stability, conformation, or localization of HS-binding proteins [9, 10, 12]. As of 2021, HS and HP were reported to bind at least 580 distinct proteins [13], a number that continues to grow as new interactions are identified.

Cell surface HSPGs function as coreceptors that facilitate receptor-ligand interactions, promote downstream signaling, and convey information from the extracellular environment into cellular responses [12]. By binding and organizing ligands, HSPGs can increase their local concentration or shape spatial gradients essential for signaling, adhesion, proliferation, differentiation, and migration. For example, numerous studies have shown that cell surface, shed, and matrix HSPGs bind chemotactic cytokines (chemokines), generating both immobilized haptotactic gradients and soluble chemotactic gradients. Chemokines comprise the most prominent group of migration guidance cues that operate in vertebrates [14]. Most chemokines possess a positively charged stretch of amino acids at their C-terminus that mediates binding to HS [15, 16]. For most chemokines, the HS-binding and receptor-binding (N-terminus) domains are spatially separated, allowing them to bind to HSPGs and their respective signaling receptors simultaneously [17]. Furthermore, at least for MCP-1 (CCL2), MIP-1 (CCL4), RANTES (CCL5), and MIP-2 (CXCL2), their HS binding mutants that retain chemotactic activity in vitro have significantly reduced capacity to induce leukocyte migration in mice [18, 19], suggesting that HSPG interactions are critical for chemokine functions in vivo. HSPGs are also major components of the endothelial glycocalyx and regulate leukocyte recruitment [20, 21]. Heparanase-mediated degradation of HS can promote leukocyte adhesion and contribute to inflammatory phenotypes, including vascular hyperpermeability and microvessel thrombosis.

Proteomic analyses have also revealed that roughly 14% of the HS/HP interactome is associated with cytokine-cytokine receptor pathways [22], emphasizing the broad role of HSPGs in cytokine-mediated immune regulation. HSPGs can both potentiate cytokine signaling and mitigate excessive inflammatory responses. For instance, given the cytotoxic effects of some interferon-stimulated genes, several suppressors and antagonists modulate IFNγ activity. HSPGs provide one such regulatory mechanism: by binding IFNγ, they retain the cytokine near its site of production and limit systemic dissemination, thereby protecting the organism from IFNγ-induced immunopathology, such as weight loss, sickness behavior, and lethal toxicity [23].

We now know that virtually all immune cells express one or several HSPGs. HSPGs have long been recognized as regulators of interactions essential for immune cell development [24, 25]. Certain HSPGs also serve as leukocyte markers, being either highly expressed on specific cell types or selectively induced during differentiation. For example, SDC1, designated CD138 in the cluster of differentiation system, is abundantly expressed on plasma cells and their malignant counterpart, myeloma cells, and is widely used as a diagnostic marker in clinical pathology [26]. Previously unrecognized roles of HSPGs in immunity are also emerging. One striking example is the discovery that granzyme K, a tryptase-like serine protease secreted by CD8 + T cells, can activate the complement cascade [27]. In this mechanism, cell surface HSPGs act as receptors for granzyme K, enhancing its ability to cleave C4 and C2 and thereby promoting assembly of membrane-bound C3 convertases. In the following sections, this review will highlight key examples illustrating the diverse roles of HSPGs in immune cell development, function, and pathology.

## 2 | HSPGs in Hematopoietic Stem Cells

Hematopoietic stem cells (HSCs) give rise to all mature blood and immune cells, and their development is tightly regulated by HSPGs. HSPGs influence multiple stages of HSC biology, including HSC migration, retention, proliferation, differentiation, and lineage commitment [28, 29], as well as the mesenchymal niche of HSCs [30]. The regulatory roles begin early during embryogenesis, when mesodermal cells differentiate into hemangioblasts and when embryonic stem cells (ESCs) give rise to HSCs. Flk1^+^ hemangioblasts, which are precursors of hematopoietic lineages, transiently express 3-*O*-sulfated HS motifs recognized by the HS4C3 antibody. These motifs decline rapidly as hemangioblasts differentiate into hematopoietic cells [31], suggesting that 3-*O*-sulfated HS domains regulate hemangioblast differentiation. Moreover, mouse ESCs lacking EXT1, the enzyme required for HS chain polymerization, fail to differentiate into hematopoietic lineages [32]. Intriguingly, supplementation with HP restores hematopoietic colony formation in *Ext1−/−* ESC cultures, but this rescue requires HP chains longer than six disaccharides and containing *N*- and 6-*O*-sulfates. Collectively, these data suggest that specific HS sulfation motifs regulate key morphogen, growth factor, and cytokine interactions essential for the generation of functional HSCs.

HSCs express several HSPGs on their cell surface, including at least SDC1, SDC2, and GPC3 [33–36]. SDC1 is expressed on approximately one-third of long-term HSCs in mice, and *Sdc1−/−* mice show a mild reduction in HSC numbers in the bone marrow [34]. Additionally, *Sdc1−/−* HSCs are functionally defective in their ability to reconstitute hematopoietic cells. Injection of *Sdc1−/−* HSCs into irradiated wild-type (Wt) mice gave significantly fewer total leukocytes and CD11b+ myeloid cells compared to reconstitution with Wt HSCs, suggesting that SDC1 promotes myelopoiesis. SDC2 is abundantly expressed in long-term HSCs, with expression levels 10-fold higher than in differentiated hematopoietic cells [36]. Isolation from bone marrow cells based solely on SDC2 surface expression enriches long-term HSCs by 24-fold, indicating that SDC2 is a specific marker for long-term HSCs. Additionally, SDC2 is strongly expressed in repopulating long-term HSCs and regulates HSC repopulating capacity by controlling HSC quiescence via CDKN1c (p57) [36]. This activity is also specific to SDC2, as expression levels of SDC1, SDC3, and SDC4 remain unchanged across hematopoietic cell populations. However, other HSPGs may regulate stem cell functions under disease conditions. For example, SDC1 promotes leukemia stem cell localization, proliferation, migration, and dissemination to distant sites in blast crisis chronic myeloid leukemia through coordinating β7 integrin functions [37]. These observations suggest that expression patterns of HSPGs might tightly control the normal and pathological phenotypes of HSCs.

Deletions or point mutations in the GPC3 gene cause the human disorder Simpson-Golabi-Behmel Syndrome (SGBS) [38, 39]. Affected individuals show pre- and postnatal overgrowth, increased susceptibility to certain pediatric cancers, and skeletal abnormalities. Interestingly, GPC3 is expressed on human and mouse HSCs, and *Gpc3−/−* mice show a selective impairment in the development of the CD11b+ and GR1+ hematopoietic lineage that gives rise to monocytes/macrophages and neutrophils [40]. How GPC3 affects the development of a specific hematopoietic lineage remains unclear, but GPC3 promotes the homing and retention of HSCs in the bone marrow by inhibiting the degradation of CXCL12, which mediates HSC migration into the bone marrow [35]. Mechanistically, GPC3 on bone marrow endothelial cells binds tissue factor pathway inhibitor (TFPI) [41], and the GPC3-TFPI complex inhibits the degradation of CXCL12 by CD26 [35]. GPC3 can also directly bind CD26 and inhibit its peptidase activity [42], suggesting that ensuring the patency of the CXCL12-CXCR4 signaling axis and subsequent homing and retention of HSCs is a major function of GPC3 in the bone marrow.

## 3 | HSPGs in B Cell Development and Function

**FIGURE 2 |:**
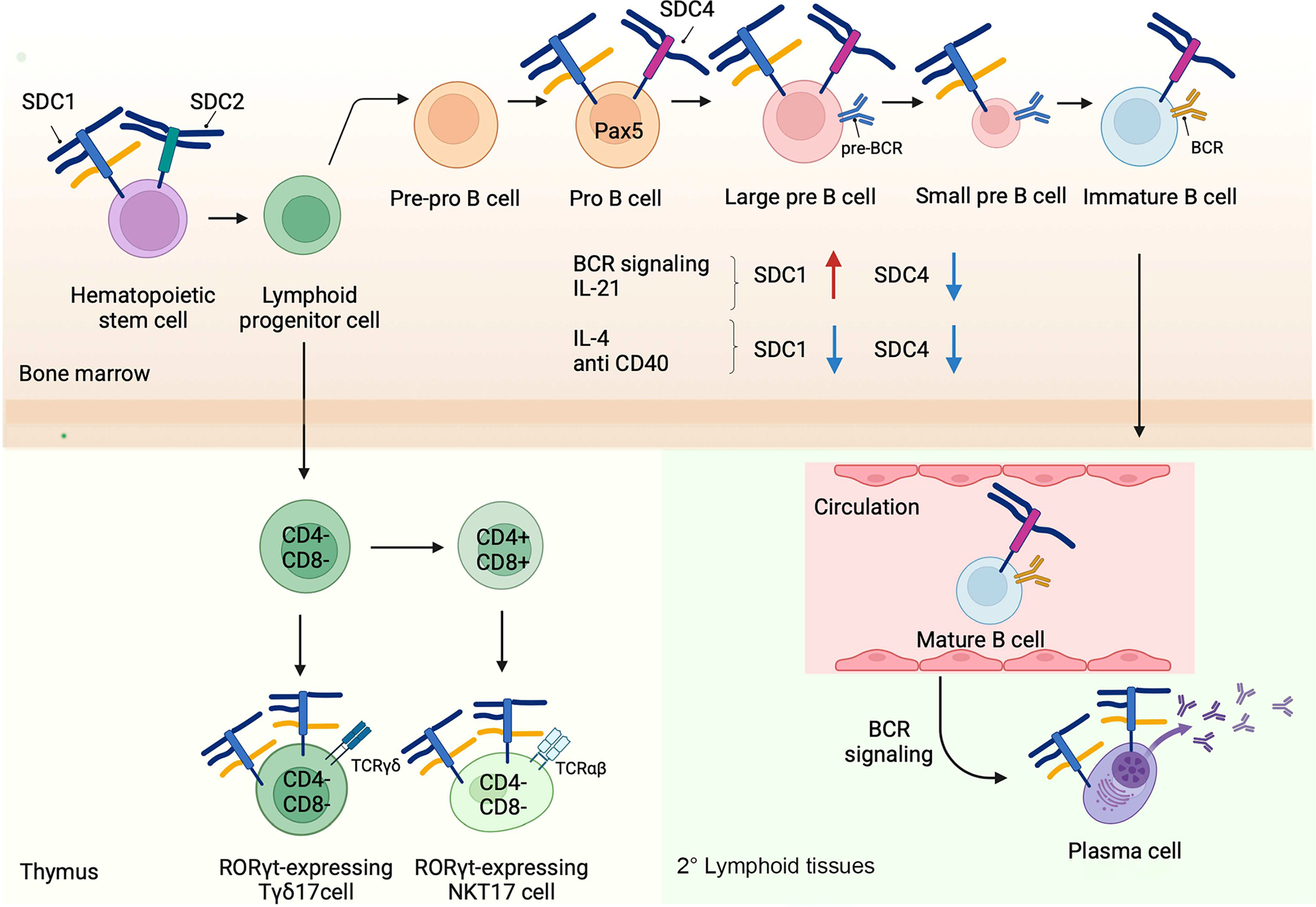
SDCs in lymphocyte development. SDC1 and SDC2 are enriched in murine HSCs compared with more differentiated hematopoietic progenitors. SDC1 is expressed on pro- and pre-B cells within the bone marrow but is absent from circulating mature B cells. During terminal differentiation into plasma cells, SDC1 is re-expressed, marking fully differentiated antibody-secreting cells. SDC4 transcripts appear in pre-pro B cells, and SDC4 HSPGs are first expressed on the cell surface at the pro-B cell stage under regulation by Pax5, a key transcription factor for B cell development. Although SDC4 expression diminishes in small pre-B cells, it re-emerges in immature and mature circulating B cells. B cell receptor (BCR) signaling alters SDC expression, further shaped by IL-4, IL-21, and CD40 ligation. During T cell development, HS is required to maintain the thymic microenvironment, ensuring proper T cell output at specific stages. RORγt-expressing T cells (*e.g*., NKT17, Tγδ17) express SDC1, although the factors inducing this expression remain unclear. The relative sizes of cells and molecules are not to scale.

Circulating B cells express HS on their cell surface (Figure 1). Available literature indicates that B cells primarily express SDCs at specific developmental stages. Expression of SDC1 on B lineage cells was first detected by immunostaining mouse intestines, where plasma cells in the intestinal lamina propria stained strongly with an antibody against SDC1 [43]. A later study showed that SDC1 is expressed on B precursor cells in the bone marrow, lost upon immature/transitional B cell exit into the circulation, and re-expressed when mature B cells are activated and differentiated into plasma cells [44] (Figure 2). An early study also showed that SDC4 is expressed on B lineage cells until mature B cells undergo immunoglobulin isotype switching [45].

Subsequent studies have detailed the expression patterns of SDCs during B cell development [46–48]. In the bone marrow, SDC1 is expressed on pro-B, large pre-B, and small pre-B cells but is lost on immature B cells before their exit into the circulation (Figure 2). SDC1 is absent from circulating mature B cells, and little to no expression is detected on class-switched or germinal center B cells. Expression, however, is strongly re-induced upon terminal differentiation into plasma cells. On the other hand, Sdc4 transcripts are detected in pre-B cell lines [49]. In the bone marrow, SDC4 expression is strongly induced at the pro-B cell stage, remains high at the large pre-B cell stage, but decreases markedly in small pre-B cells (Figure 2). Expression is restored in immature B cells and maintained in circulating naïve B cells but is substantially reduced in class-switched B cells and absent in germinal center B cells and plasma cells [45, 46] (Figure 2). Collectively, these findings indicate that SDC1 displays a concave-up expression pattern during B cell development—lost in the mid to late stages but sharply upregulated in plasma cells—whereas SDC4 expression fluctuates throughout development and progressively declines toward terminal differentiation.

Despite its distinctive expression pattern, CRISPR–Cas9 knockout studies have shown that SDC1 deletion has no major impact on B cell development in the bone marrow or on antigen-driven terminal differentiation into plasma cells [46]. In contrast, SDC4 deletion results in a reduction of B cells during the transition from pre-pro- to pro-B cells, which, as aforementioned, is the developmental stage at which SDC4 expression is normally upregulated. This early reduction in B cell number in *Sdc4−/−* mice is subsequently offset by a gradual recovery during the later stages of B cell maturation [46], suggesting that while SDC4 promotes progression from the pre-pro- to pro-B cell stage, it may negatively regulate the transition from pre-B to mature B cells in the bone marrow.

The mechanisms regulating syndecan expression during B cell development remain incompletely understood. Current evidence indicates that Pax5, a key transcription factor in B cell commitment, induces SDC4 expression [50] while repressing SDC1 [51]. Pax5 may therefore be a critical determinant of the progressive downregulation of SDC1 from pro- to immature B cells, and the induction and maintenance of SDC4 expression throughout early B cell development in the bone marrow (Figure 2). SDC expression is also dynamically modulated by B cell receptor (BCR) signaling [46]. Using isolated B cells from transgenic mice expressing BCR specific for hen egg lysozyme, antigen stimulation induces SDC1 expression, which is further increased by IL-21 but inhibited by IL-4 or anti-CD40. Consistent with this, IL-4 suppresses the appearance of SDC1+ follicular B cells in splenocyte cultures [52], indicating that IL-4 is a negative regulator of SDC1 expression in mature B cells. On the other hand, BCR activation downregulates SDC4 expression, an effect partially reversed by IL-4, anti-CD40, and to a lesser extent by IL-21, indicating that BCR activation and associated cytokines reciprocally regulate SDC1 and SDC4 expression. Interestingly, IL-21 binding to B cells is largely mediated by cell surface HSPGs [53]. The responsiveness of B cells to IL-21 depends on the presence of *N-*sulfated motifs in HSPGs, where low levels of *N*-sulfation correlate with markedly reduced IL-21 binding and signaling [53]. NDST1, the primary enzyme responsible for *N*-deacetylation and *N*-sulfation of HS, is down-regulated in germinal center B cells but upregulated in plasma cell precursors, consistent with IL-21-driven induction of SDC1 during plasma cell differentiation. Together, these studies demonstrate that B cells use both select HS modifications and differential expression of SDC core proteins during development to adapt to the dynamic immune environments.

IL-7 is another HS-binding cytokine that plays an essential role in early B cell development [54]. IL-7 binds to HS [55, 56], and both B-lineage precursor cells and bone marrow stromal cells use cell surface HSPGs to capture IL-7 [57]. Addition of excess HP competitively inhibits IL-7-induced proliferation of bone marrow cells and lymphoid cell lines, but not proliferation driven by IL-3, G-CSF, or GM-CSF. Likewise, enzymatic removal of cell surface HS by heparinase III impairs IL-7 binding and suppresses IL-7-dependent growth in B precursor cell lines. Together, these findings indicate a direct role for cell surface HSPGs in regulating IL-7-dependent stages of B cell development. HSPGs on stromal cells serve as reservoirs that control IL-7 bioavailability, while HSPGs on B precursor cells function as IL-7 coreceptors. Interestingly, when non-adherent bone marrow cells from *Sdc1−/−* or *Sdc4−/−* mice were cultured in the presence of IL-7, the total number of pre-pro and immature B cells increased more than 10-fold [46]. The stromal HSPG providing IL-7 is likely SDC4, since stromal cells typically lack SDC1 expression under resting conditions, whereas the IL-7 coreceptor on B precursor cells may be either SDC1 or SDC4.

Although B cell development proceeds normally in the absence of SDC1, and *Sdc1−/−* B cells can differentiate into plasma cells, these mice exhibit impaired long-term humoral immunity [47]. Consistent with the approximately 1000-fold induction of SDC1 during plasma cell differentiation [44], this phenotype is attributed to the loss of SDC1-mediated survival signaling provided by HS-binding cytokines, such as IL-6 [58] and APRIL (a proliferation-inducing ligand) [59], both of which are critical for plasma cell longevity [47]. Moreover, SDC1 levels influence plasma cell competition and retention within the bone marrow niche [60]. Long-lived plasma cells form clusters that constitute specialized survival niches in the bone marrow, where SDC1 regulates their adhesion and motility on fibronectin and promotes their clustering. Survival within these clusters depends on APRIL, to which SDC1 binds and promotes prosurvival signaling. Surface levels of SDC1 determine niche occupancy between nascent and mature plasma cells, with mature plasma cells preferentially retained due to their considerably higher SDC1 expression.

The expression pattern of SDC1 during B cell development suggests that SDC1 is primarily expressed when B precursor cells and plasma cells reside in an adherent environment. An exception is multiple myeloma, in which circulating myeloma cells also express high levels of SDC1 at the cell surface [61, 62]. SDC1 expression is polarized on myeloma cells [63], suggesting that SDC1 may regulate not only proliferation and survival, but also the migration of these tumor cells. Indeed, SDC1 expression inhibits myeloma cell invasion into collagen [64], and SDC1 is a coreceptor for factors that regulate these cellular processes, including FGFs, HGF, BMPs, APRIL, and IL-6 [59, 65–67]. SDC1 expression and shedding are both adverse prognostic factors in multiple myeloma [68]. Additionally, SDC4 is expressed on B lymphomas, but not on myeloma cells [45], suggesting that SDC1 mediates functions specific to myeloma progression. These observations suggest that SDC1 is a prime target for multiple myeloma therapy. Accordingly, anti-SDC1 antibody-drug conjugates (ADCs) [69] and chimeric antigen receptor-T (CAR-T) cells targeting SDC1 [70] have been developed and evaluated in clinical studies. For example, an anti-SDC1 chimerized monoclonal antibody conjugated to maytansinoid DM4 (Indatuximab ravtansine, BT062) produced limited clinical responses as monotherapy in a phase I/II trial [71], whereas its combination with lenalidomide or pomalidomide increased overall response rates to 85%–88% [72]. A clinical trial of anti-SDC1 CAR T-therapy (ATLCAR.CD138) in patients with relapsed and refractory multiple myeloma is currently ongoing (NCT03672318).

Additionally, most, if not all, of the pathologic activities of myeloma SDC1 are mediated by HS, and heparanase critically regulates multiple myeloma progression [73–75]. Heparanase promotes myeloma growth, angiogenesis, and metastasis, and can also prime exosome and autophagosome biogenesis and enhance myeloma chemoresistance [73, 74, 76]. Heparanase-induced SDC1 shedding contributes to the pathogenesis and progression of myeloma and several other cancers [77, 78], and heparanase can mediate chemotherapy-induced macrophage activation that supports tumor growth [79]. Consistent with these findings, heparanase-inhibiting compounds and neutralizing antibodies attenuate myeloma growth and metastasis [80–82], suggesting that heparanase and heparanase-mediated pathways are viable therapeutic targets in multiple myeloma.

## 4 | HSPGs in T Cell Development and Function

HSPGs regulate T cell development, maturation, and function. In the thymus, HS is most abundantly expressed on thymic fibroblasts compared with endothelial, epithelial, and hematopoietic cells [83]. Conditional deletion of HS in fibroblasts by crossing *Ext1*(fl/fl) mice with *Pdgfrα-Cre* mice resulted in embryonic lethality, like global *Ext1* deletion. Although T cell development appeared normal in fetal thymic organ cultures from these conditional knockout mice, thymus size was reduced, and T cell output was decreased, indicating that HSPGs on thymic fibroblasts are required for thymus organogenesis and optimal T cell development. Binding of the homeostatic chemokines CCL19, CCL21, and CXCL12 was reduced in HS-deficient thymic fibroblasts compared with Wt fibroblasts, leading to diminished CCL19/CCL21 gradients and decreased infiltration of bone marrow-derived dendritic cells (DCs) into fetal thymic slices [83]. These data suggest that HSPGs on thymic fibroblasts ensure proper positioning of thymocytes and DCs in the thymic medulla, thereby permitting normal T cell selection and maturation. In addition, sulfated domains in HS are important: conditional KO mice lacking NDST1 or NDST2 in T cells (*Ndst1*(fl/fl) or *Ndst2*(fl/fl) crossed with *Lck-Cre*) also display mislocalized T cells in lymphoid organs despite largely normal T cell development. Interestingly, splenic T cells from mice lacking both NDST1 and NDST2 show enhanced proliferation in response to anti-CD3 stimulation, suggesting that sulfated HS on T cells restrains cytokines and growth factors that drive proliferation of activated T cells [84].

The importance of HSPGs in T cell development in the thymus was further substantiated by a clinical study investigating the basis of a primary T cell immunodeficiency in three patients from two families [85]. The genetic defect was traced to homozygous missense mutations in EXTL3, an *N*-acetylglucosaminyltransferase crucial for initiating HS biosynthesis. Inactivation of *Extl3* in zebrafish reproduced the thymopoiesis defect, which was rescued by injection of Wt human *Extl3* RNA, consistent with the high degree of conservation (> 95%) of the catalytic domain between human and zebrafish EXTL3. Differentiation studies using patient-derived induced pluripotent stem cells (iPSCs) indicated that EXTL3 mutations cause T cell immunodeficiency by directly impairing the proliferative capacity of lymphohematopoietic progenitor cells and disrupting thymic epithelial progenitor cell differentiation [85]. Together, these data indicate that HSPGs expressed by progenitor cells, thymic epithelial cells, and thymic fibroblasts exert a strong influence on T cell development in the thymus. Future studies are expected to define the specific functions of HSPGs elaborated by different cell types in the thymic microenvironment during T cell development.

Circulating, mature T cells express HS on their cell surface (Figure 1). Although the expression of HSPGs on mature T cells has been reported in numerous studies, the identities of the specific T cell HSPG core proteins have not been clearly established in most cases [86–94]. SDC1 has been proposed to be expressed on peripheral CD4^+^ T cells [95], but because SDC1 is a specific marker of both circulating and bone marrow plasma cells as well as myeloma cells, its expression on circulating T cells is likely to be very low. Indeed, although SDC1, SDC3, and SDC4 mRNA can be detected in resting CD4+T cells, little to no SDC1 protein is found on resting or activated CD4 + T cells. These findings indicate that the HSPGs recognized by 10E4 antibodies on circulating T cells are likely core proteins other than SDC1 (Figure 1).

An exception is MRL/lpr mice, which carry a Fas mutation and harbor a large population of SDC1+T cells [96]. MRL/lpr mice spontaneously develop a systemic lupus erythematosus (SLE)-like disease where high levels of autoantibodies directed against nuclear antigen cause damage. Interestingly, soluble SDC1, proteolytically released from SDC1 + T cells, binds APRIL and enhances APRIL-mediated plasma cell generation and autoreactive antibody production [97]. Among splenic CD3 + T cells in MRL/lpr mice, approximately 28% are SDC1+ [96]. These findings raise the intriguing possibility that a substantial population of T progenitor cells may be SDC1+ until removed by Fas-mediated apoptosis during negative selection in the thymus, although the function of SDC1 in T cell development remains unknown. One speculative idea is that SDC1 might cooperate with Fas or FasL to eliminate autoreactive T cells, but this would be at odds with the generally anti-apoptotic role of SDC1 [47, 98]. By contrast, only a small fraction of resting CD3+T cells express SDC4 [45], whereas SDC4 is strongly upregulated upon CD4^+^ T cell activation [99], suggesting that SDC4 expression in T cells is activation dependent.

Studies over the last decade have established that two relatively rare T cell subsets, NKT17 cells [100–104] and Tγδ17 cells [105, 106], express SDC1 on their surface. NKT17 cells develop from CD4+CD8+ double-positive thymocytes [104], whereas Tγδ17 cells arise from CD4−CD8− double-negative thymocytes in the thymus [107] (Figure 2). The precise developmental stage at which these T cells acquire SDC1 expression is not known, but it occurs after lymphoid progenitor cells reach the thymus, because most SDC1+ lymphoid cells in the bone marrow are B cell precursors that express B220 [44]. A defining feature of both NKT17 and Tγδ17 cells is their production of IL-17. However, expression of the nuclear hormone receptor RORγt, the master transcription factor that drives IL-17 production, is not sufficient to induce SDC1, since forced expression of RORγt in NKT1 and NKT2 cells does not upregulate SDC1 [103], and Th17 cells, another IL-17-producing, RORγt+ subset, also lack SDC1 [105]. It is not yet known whether type 3 innate lymphoid cells (ILC3s), which express RORγt and secrete large amounts of IL-17, express SDC1. Because mature Tγδ17 cells are CD4−CD8− and the majority of mature NKT17 cells are also CD4−CD8−, whereas Th17 cells are CD4+, transcriptional programs that regulate CD4 expression may inversely regulate SDC1 expression in T cells. For example, RUNX family members and AP4 silence CD4 in T cells [108], whereas these transcription factors activate SDC1 expression in various cell types [109–111]. However, the effects of these transcription factors on SDC1 expression in NKT17 and Tγδ17 cells are not known.

SDC1 + NKT17 cells have been identified in multiple tissues, including the thymus, visceral adipose tissues, spleen, lung, and peripheral lymph nodes [100, 102, 103]. The frequency of SDC1 + NKT17 cells among total NKT cells is high in white adipose tissues, peripheral lymph nodes, and lung (approximately 60%, 20%, and 20%, respectively) compared with approximately 3% in the spleen and less than 1% in the thymus [100, 102]. Notably, SDC1 is not required for NKT17 differentiation because IL-17-producing, RORγt-positive NKT17 cells develop normally in the thymus of *Sdc1−/−* mice [100, 103]. However, thymic NKT17 cell numbers are significantly increased in *Sdc1−/−* mice in one study [100] and modestly increased in another study [103], both at the expense of NKT1 cells. *Sdc1−/−* and Wt NKT17 cells are activated similarly by alpha-galactosylceramide (αGalCer), a prototypic glycolipid antigen of NKT cells [100, 103], and SDC1 deficiency does not alter IL-17 production in NKT17 cells stimulated with PMA and ionomycin [103]. These observations suggest that while SDC1 is dispensable for NKT17 development and acute activation, it negatively regulates NKT17 homeostasis, likely by constraining their proliferation and/or maintenance. How SDC1 accomplishes this remains unclear, and it appears counterintuitive to the usual role of cell surface HSPGs as enhancers of growth factor and cytokine signaling [12]. Because αGalCer and PMA/ionomycin are nonphysiological agonists, SDC1 may instead modulate NKT17 activation by endogenous and microbial glycolipid antigens and HS-binding cytokines, such as IL-12, and in the maintenance of NKT17 cells by HS-binding IL-7 [57, 104, 112, 113].

SDC1 expression on Tγδ17 cells has been reported in the thymus, lymph nodes, and skin [105]. Global deletion of SDC1 in mice leads to increased proliferation, decreased apoptosis, and higher frequency and absolute numbers of Tγδ17 cells in these tissues [105]. Like its role in NKT17 cells, these findings suggest that SDC1 acts as a negative regulator of Tγδ17 cell growth, maintenance, and survival. In an imiquimod-induced psoriatic dermatitis model, *Sdc1−/−* mice showed increased numbers of Tγδ17 cells in draining lymph nodes, along with greater ear swelling and epidermal thickening compared to Wt controls [105]. Comparable exacerbation of disease was observed when TCRβδ KO mice were reconstituted with *Sdc1−/−* T cells [105], indicating that loss of SDC1 specifically in T cells, rather than in non-T cells, underlies the heightened susceptibility to imiquimod-induced psoriatic dermatitis. Together, these results support a model in which SDC1 suppresses excessive activation and proliferation of Tγδ17 cells in IL-17-driven diseases such as psoriasis, most likely by promoting Tγδ17 cell apoptosis. These findings also underscore the need to define whether SDC1 exerts overlapping regulatory functions in NKT17 and Tγδ17 cells, as they both modulate host responses in infectious and immune-mediated disorders.

HSPGs expressed by non-immune cells also modulate T cell activity. Cell surface HSPGs on endothelial and epithelial cells bind chemokines and form haptotactic chemokine gradients to guide the directional migration of leukocytes [16, 114, 115]. In contrast, soluble HS and HP can inhibit T cell migration in response to chemokines. For example, in an air pouch inflammation model, HP oligosaccharides inhibited CXCL9-mediated recruitment of CD4+T cells when preincubated with CXCL9, presumably by blocking CXCL9 interactions with endogenous cell surface HSPGs [116]. Likewise, excess HS inhibits T cell migration toward CCL11 and CCL17 [117], chemokines implicated in Th2 inflammatory diseases such as asthma. The cellular source of soluble HSPG is not fully defined, but cell surface HSPGs such as SDC1 are shed from epithelial cells under inflammatory conditions [10, 118, 119]. Consistent with a protective role for shed SDC1, *Sdc1−/−* mice are hypersusceptible to allergic lung disease triggered by *Aspergillus fumigatus* extracts [117]. Allergen-challenged *Sdc1−/−* mice show increased airway hyperresponsiveness, eosinophilia, and glycoprotein secretion compared with Wt mice, and these phenotypes are mitigated by administration of purified, native SDC1 ectodomains but not by SDC1 core proteins without HS, implicating HS chains on shed SDC1 as critical regulators of chemokine-driven Th2 cell recruitment.

The oxazolone-mediated contact allergy mouse model is a model of delayed-type hypersensitivity (DTH), in which activated T cells drive an inflammatory response that mimics human allergic inflammation. Deletion of SDC1 in mice prolongs the DTH reaction, with increased leukocyte influx and elevated expression of proinflammatory cytokines and chemokines [120]. These phenotypes were attributed to the loss of SDC1-mediated inhibition of interactions between leukocyte β2 integrins and ICAM-1. Similarly, in a Gram-positive toxic shock model, in which bacterial superantigens trigger a systemic inflammatory response syndrome, *Sdc1−/−* mice show increased T cell accumulation in the liver, a systemic cytokine storm, and mortality compared with Wt mice [121]. Here, SDC1 ectodomains shed from hepatocytes attenuate disease by inhibiting recruitment of activated T cells, as SDC1 deficiency does not impair the capacity of superantigen-activated T cells to produce cytokines. Tumor cell SDC1 also appears to inhibit anti-tumor T cell responses [122]. Pharmacologic inhibition or genetic deficiency of SDC1 increases CD8+T cell infiltration into tumors and augments tumor antigen presentation and susceptibility to T cell-mediated killing. Notably, SDC1 inhibition enhances the therapeutic efficacy of anti-PD-1 treatment in suppressing tumor growth [122], suggesting that targeting SDC1 may improve responses to immune checkpoint blockade.

## 5 | HSPG Regulation of Neutrophil Functions

Neutrophils are typically the first leukocytes recruited to sites of injury and infection, and they promote tissue repair and eliminate pathogens through multiple mechanisms. Deficiencies in neutrophils, either inherited or acquired, often result in severe infections [123, 124]. Neutrophils express HSPGs on their cell surface [125, 126] (Figure 1) and within granules [127–129]. Surface HSPGs of neutrophils bind neutrophil elastase and cathepsin G, which help localize these enzymes at the plasma membrane and modulate their activity [125]. Cell surface HSPGs also act as coreceptors for ELR+CXC chemokines, synergizing with GPCRs to enhance neutrophil migration [126]. Intracellular HSPGs in azurophilic granules bind antimicrobial proteases, such as elastase and cathepsin G, and maintain them in an inactive state [130]. Upon neutrophil activation, a K+ flux releases the proteases from HSPGs during degranulation and activates them. This mechanism is important in neutrophil-mediated defense against *Staphylococcus aureus* and *Candida albicans*, compared with killing mediated by reactive oxygen species (ROS) [130]. Because neutrophil elastase also plays a key role in defense against Gram-negative bacteria, including *Escherichia coli* and *Klebsiella pneumoniae* [131, 132], intracellular HSPGs likely help coordinate microbial killing while minimizing collateral protease-mediated tissue injury. Moreover, conditional inactivation of *Hs2st1* in neutrophils markedly reduces neutrophil extracellular trap (NET) formation and increases susceptibility to group B *Streptococcus* sepsis [133]. Although *Hs2st1* deletion does not affect protease secretion, elastase is crucial for NET formation, and elastase-deficient mice fail to produce NETs during *K. pneumoniae* lung infection [134]. These results suggest that 2-*O*-sulfated motifs in intracellular neutrophil HSPGs may be required for elastase-driven chromatin decondensation, an early and vital step in NETosis.

HSPGs expressed by non-immune cells also regulate neutrophil activation, recruitment, and antimicrobial functions. Endothelial HSPGs capture and immobilize chemokines on the luminal surface, presenting chemokines to circulating leukocytes for adhesion and activation [16, 135–137]. HS binding enhances IL-8 (CXCL8) activity [138, 139], and endothelial HSPGs interact with MIP-2 to generate chemotactic gradients that direct neutrophil migration [140]. HSPG interactions also shape the in vivo specificity and function of neutrophil-active chemokines [141, 142]. The mechanistic basis for this specificity remains incompletely understood, but HS sulfation patterns appear critical. For instance, endothelial *Hs2st1* deletion enhances neutrophil infiltration in a thioglycolate-induced peritonitis and dorsal air pouch models by increasing IL-8 and MIP-2 retention at the cell surface [143], suggesting that 2-*O*-sulfates modulate chemokine binding. On the other hand, the same *Hs2st1* KO mice show decreased neutrophil trafficking to the liver in both sterile and infectious liver injury [144]. Furthermore, heparanase treatment promotes neutrophil adhesion and transmigration across the endothelial barrier [145]. These findings suggest that the effects of HS 2-*O*-sulfation on neutrophil recruitment might depend on the disease context, and that changes in HS within the endothelial glycocalyx dynamically control neutrophil recruitment. Alternatively, because SDC2, SDC3, and SDC4 are expressed by endothelial cells and bind neutrophil-active chemokines [146–148], HS modifications on HSPGs expressed in different cellular compartments may differentially regulate neutrophil-chemokine interactions. Regardless, other neutrophil chemoattractants, such as formylated peptides, leukotrienes, C5a, and platelet-activating factor, do not appear to engage HSPGs, underscoring the specificity of HSPG-chemokine interactions in neutrophil recruitment.

HSPGs also contribute to the resolution of neutrophilic inflammation. *Sdc1−/−* mice display heightened neutrophilic inflammation, multi-organ injury, and increased mortality compared with Wt controls during endotoxic shock [149]. Although the overall magnitude of systemic inflammation was comparable between endotoxemic *Sdc1−/−* and Wt mice, tissue-bound KC (CXCL1) and MIP-2 persisted at higher levels in *Sdc1−/−* mice at later time points after LPS challenge. LPS-induced KC and MIP-2 mRNA expression and splenocyte chemokine protein production were similar across genotypes, indicating that differences in chemokine clearance, not synthesis, account for the phenotypes. In Wt mice, SDC1 shedding closely correlated with the removal of tissue-bound chemokines, suggesting that activation of SDC1 shedding facilitates the resolution of immobilized chemokine gradients. Consistent with this mechanism, peptide hydroxamate inhibition of SDC1 shedding exacerbated disease by preventing chemokine clearance and prolonging neutrophil inflammation, whereas administration of exogenous HS reduced tissue-bound chemokine levels and accelerated resolution of accumulated neutrophils in endotoxemic *Sdc1−/−* mice [149]. Because SDC1 is not expressed on endothelial cells, these findings imply that HS chains of SDC1 ectodomains bind and remove chemokines tethered to endothelial HSPGs, thereby promoting the resolution of neutrophilic inflammation.

**FIGURE 3 |:**
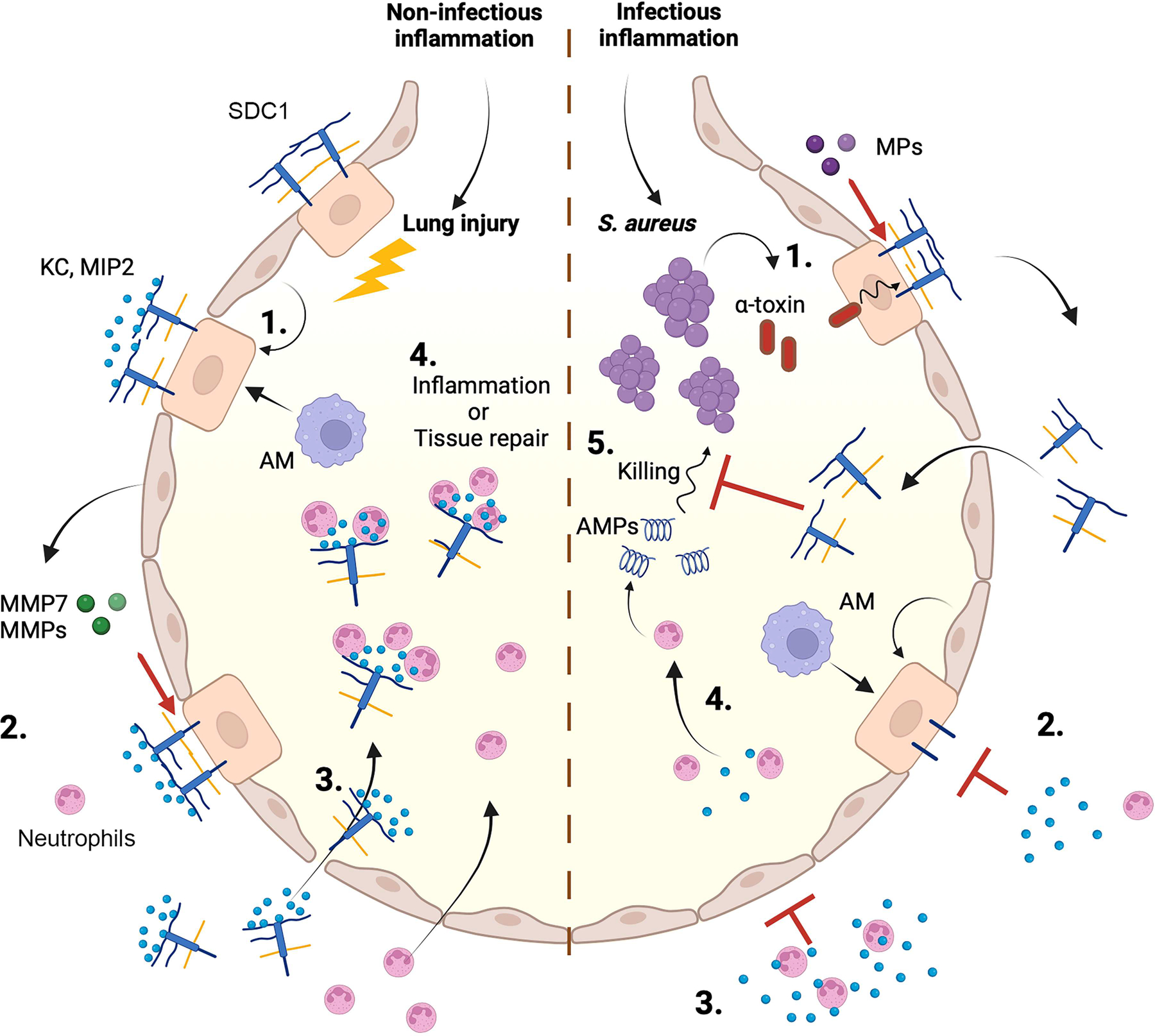
Functions of SDC1 in sterile and infectious lung inflammation. Non-infectious inflammation: (1) Lung injury induces chemokines, MMPs, and other pro-inflammatory mediators in epithelial cells and alveolar macrophages (AMs). (2) Chemokines such as KC and MIP2 bind to SDC1 HS. The SDC1/chemokine complex is then shed by MMPs into the alveolar space. (3) The shed SDC1/chemokine complex establishes a chemotactic gradient that guides the transepithelial migration of neutrophils into the alveolar compartment. (4) Neutrophil accumulation in the alveolar space promotes either tissue repair or progression of inflammation. Infectious inflammation: (1) *S. aureus* α-toxin binds to receptors (*e.g*., β1 integrins, ADAM10) on alveolar type II epithelial cells and triggers premature SDC1 shedding. SDC1 ectodomains lacking chemokine cargo are released into the alveolar space. (2) Chemokines are induced, but with SDC1 depleted, newly synthesized chemokines remain unbound. (3) Neutrophils are recruited to the lung interstitium, but in the absence of a SDC1/chemokine gradient, transepithelial migration is delayed. (4) Neutrophils in the lung interstitium enter the alveolar space randomly. (5) Neutrophil-mediated host defense is further compromised when SDC1 ectodomains inhibit antimicrobial peptides (AMPs) in an HS-dependent manner. The relative sizes of cells and molecules are not to scale.

While much is known about how endothelial HSPGs immobilize and present chemokines to circulating leukocytes, far less is understood about the role of epithelial HSPGs in regulating chemokine presentation and localization during an inflammatory response. In polarized epithelial cells, chemokines such as IL-8 are preferentially secreted basolaterally [150–153], yet they accumulate on the apical surface [150], indicating transepithelial transport to establish chemokine gradients. In a mouse model of bleomycin-induced lung injury, SDC1 was shown to mediate this process [154]. Upon injury, chemokines such as KC are induced and bind to SDC1 on the basolateral surface of alveolar type II (ATII) epithelial cells (Figure 3). Concurrently, matrix metalloproteinase-7 (MMP7), a known SDC1 sheddase, is upregulated. In the absence of MMP7, levels of both KC and SDC1 in the alveolar compartment remain low, and neutrophils are confined to the interstitium of injured lungs, unable to migrate across the epithelial barrier. Likewise, loss of SDC1 reduces KC levels in the alveolar space and impairs neutrophil transepithelial migration. Together, these findings indicate that MMP7-dependent shedding of the SDC1-KC complex generates a transepithelial chemokine gradient that directs neutrophil recruitment to sites of alveolar epithelial injury (Figure 3). Moreover, this mechanism functions as a checkpoint that restricts neutrophil activation at sites of epithelial injury [155].

Interestingly, *Sdc1* deletion is a gain-of-function mutation in several models of bacterial infection [156–163], suggesting that pathogens may subvert SDC1 shedding to dysregulate neutrophil recruitment to sites of infection. Indeed, several microbial pathogens can trigger SDC1 shedding, either directly with their sheddases or indirectly by activating the host’s shedding machinery [157, 160, 163–169]. For example, *S. aureus* α-toxin can rapidly and potently induce SDC1 shedding by binding to a receptor on epithelial cells and activating a protein tyrosine kinase (PTK)-dependent signaling mechanism that triggers MMP-mediated shedding at the cell surface [168]. If this occurs before chemokine induction and loading onto SDC1 HS, it could disrupt chemokine transport into the alveolar space, delay neutrophil recruitment, promote disorganized neutrophil migration into the alveolar compartment, and permit bacterial proliferation (Figure 3). Alternatively, loss of SDC1 shedding may simply reduce chemokine accumulation in the alveolar space during infection, resulting in fewer neutrophils and a weaker inflammatory response. Because one of the hallmarks of *S. aureus* pneumonia is an intense neutrophilic inflammatory response that contributes to lung injury [170, 171], deletion of SDC1 may improve disease outcome by preventing exaggerated neutrophilic inflammation. Consistent with this idea, mice infected with α-toxin-deficient *S. aureus* exhibit significantly lower levels of KC and MIP-2 in the airways, without changes in chemokine mRNA levels, along with a concomitant decrease in neutrophil numbers in the airways and lung tissue [172]. Whether this indicates decreased SDC1 shedding activation caused by the α-toxin-deficient *S. aureus* strain remains to be determined.

Bacterial pathogens also exploit SDC1 ectodomains’ ability to inhibit neutrophil-mediated host defense to promote pathogenesis. For example, SDC1 ectodomains inhibit *Pseudomonas aeruginosa* and *S. aureus* killing by cationic antimicrobial peptides [156, 159] (Figure 3). Specifically, SDC1 ectodomains suppress the antibacterial activity of neutrophil-derived CRAMP (cathelicidin-related antimicrobial peptide) by binding the peptide through 2-*O*-sulfated HS domains and interfering with CRAMP binding to bacterial targets [159]. Other cathelicidins, such as human LL-37, are also inhibited by HS and HP compounds containing 2-*O*-sulfates, but not by those lacking this modification [173], suggesting that SDC1 ectodomains may broadly inhibit cathelicidin-mediated host defense in infections where these peptides are important. Additionally, SDC1 ectodomains inhibit NET-mediated killing of *L. monocytogenes* in vivo by interfering with NET formation and/or promoting NET disintegration [163]. Soluble HS also inhibits neutrophil elastase activity [138, 174], suggesting that shed HSPGs such as SDC1 ectodomains may impair elastase-dependent antimicrobial defenses. More recently, soluble HS was shown to inhibit neutrophil killing of *C. albicans* in vitro and in a vulvovaginal candidiasis model [175], extending these mechanisms to fungal infection. Altogether, these findings highlight shed HSPG-mediated suppression of neutrophil function as a potentially broad immune-evasion strategy used across diverse pathogens in different tissues.

## 6 | HSPGs in Dendritic Cells

The primary function of DCs is to sample pathogens, cancer cells, or foreign substances, process their antigens, and present the fragmented antigens on MHC molecules for T cell recognition and activation [176]. DCs are activated and mature upon encountering antigens, then travel to lymph nodes to present antigens to T cells. DCs express several SDCs and GPCs on their cell surface [177]. Monocyte-derived DCs, bone marrow-derived DCs (BMDCs), tumor-associated DCs, and Langerhans cells of the epidermis express at least SDC1, SDC3, SDC4, GPC1, GPC2, GPC3, and GPC5 [178, 179]. Expression of HSPGs on DCs depends on their activation and differentiation status. For example, immature DCs mainly express GPC1, whereas mature DCs express GPC3 [177]. In monocyte-derived DCs and Langerhans cells, SDC4 is significantly upregulated within the first few hours of LPS-induced DC maturation, accompanied by a notable downregulation of SDC1 [179]. SDC1 downregulation is suppressed by SDC4 knockdown, indicating an inverse mechanistic relationship between SDC1 and SDC4 expression in DCs. On the other hand, although SDC4 induction is most prominent during BMDC maturation, SDC1 and SDC3 are also induced during LPS-induced BMDC maturation [178], which suggests that SDC expression may be regulated differently across DC subtypes.

SDC4 inhibition, knockdown, or knockout results in decreased DC maturation and motility, indicating that higher SDC4 levels have functional effects in DCs [178, 179]. Indeed, SDC4 promotes DC spreading on fibronectin [179], suggesting that SDC4-dependent ECM interactions support DC motility. SDC4 also promotes CCL21-mediated DC migration, likely by acting as a coreceptor for this chemokine. Sulfated domains in SDC4 HS are crucial for these processes, as DCs from NDST1-deficient mice exhibit phenotypes similar to those of *Sdc4−/−* mice [178]. However, the mechanism by which increased SDC4 expression influences DC activity remains unclear. The increased amount of SDC4 over time, differences in the fine structure of SDC4 HS, how SDC4 selectively regulates its binding partners, or a combination of these mechanisms may contribute to how SDC4 prominently affects DC functions. For example, SDC4 deletion reduces CCR7 expression on DCs, possibly by altering CCR7 recycling and surface expression [180]. Because exogenous HS can promote the maturation of DCs [181], HS chains of shed SDC4 ectodomains may function similarly. Alternatively, since SDC1 is simultaneously downregulated during SDC4 induction and *Sdc1−/−* DCs migrate faster to CCL21 and CCL19 [182], SDC4 may indirectly influence DC activities by promoting SDC1 downregulation. Regardless of the mechanism, the positive effect of SDC4 induction on DC maturation and motility is believed to contribute to the development and sustenance of primary immune responses.

SDC4 induction in DCs can also have pathogenic effects depending on the disease context. For instance, in a mouse model of Lewis lung carcinoma, tumor growth is significantly attenuated in *Sdc4*/*Ndst1−/−* double KO mice. In this model, SDC4 and NDST1 deficiency enhances DC maturation yet paradoxically impairs DC trafficking to draining lymph nodes [178, 183]. Similarly, in ovalbumin-induced allergic lung inflammation, SDC4 deletion or treatment with anti-SDC4 polyclonal antibodies impairs the directional migration of antigen-presenting DCs, thereby reducing overall lung inflammation [180]. Beyond their role in inflammation, HSPGs on DCs are frequently exploited by pathogens. In sexually transmitted HCV, high SDC4 expression on activated mucosal Langerhans cells facilitates viral transmission, which can be abrogated by SDC4 silencing or inhibiting HS interactions [184]. SDC3 plays a parallel role in HIV-1 infection, serving as the primary adhesion receptor on DCs [185]. These findings suggest that microbial pathogens utilize HSPGs on DCs as coreceptors or primary entry points, mirroring the mechanisms used by viral pathogens to infect epithelial cells [186]. While anti-SDC4 antibodies are used in several studies to demonstrate the importance of SDC4, a mechanistic nuance remains: SDC4 functions are largely mediated by its HS chains, yet these antibodies target the core protein. It is possible that core protein binding exerts steric hindrance on HS-ligand interactions, though an alternative possibility that these antibodies are simply immunodepleting SDC4+ DCs cannot be ruled out.

## 7 | HSPGs in Macrophages

Various HSPGs, including SDCs, GPCs, and perlecan, are expressed by macrophages and regulate polarization and cellular function. For instance, *Sdc1* mRNA is markedly elevated in thioglycolate-elicited macrophages, and protein levels are further regulated by a cAMP-dependent post-transcriptional mechanism [187], suggesting a distinctive control of SDC1 expression. Expression of SDC2 and SDC4 has also been reported in activated macrophages [188] and in the macrophage-like cell line P388D1 [189]. Functionally, SDC1 expression correlates with M2 macrophage polarization and high intrinsic motility, as SDC1-deficient macrophages exhibit impaired migration and enhanced adhesion [190]. On the other hand, there is a population of SDC1+ tumor-associated macrophages characterized by their proinflammatory phenotypes [191]. The SDC1+ macrophage population is significantly increased in both pancreatic ductal adenocarcinoma patients and mouse models. Mechanistically, SDC1 on the tumor-associated macrophages promotes tumor progression by enhancing IL-34 signaling and establishing a feedforward loop with immunosuppressive Siglec F+ neutrophils [191].

GPC4 has also been implicated in promoting M2 polarization [192], although whether SDC1 and GPC4 have distinct or overlapping roles in macrophage differentiation remains unknown. Beyond polarization, macrophage HSPGs participate in host-pathogen and metabolic interactions—for example, serving as receptors for HIV-1 [193] or low-density lipoprotein (LDL). Cell surface HSPGs also modulate macrophage activation state by maintaining type I interferon signaling in a quiescent state through sequestration of IFNβ [194]. Collectively, these findings highlight macrophage HSPGs as multifaceted regulators of cell behavior and immune signaling, with many of their molecular interactions yet to be defined.

### 7.1 | Serglycin in Mast Cells and Other Leukocytes

Mast cells are among the first responders to infectious agents and toxins and are also key mediators of allergic responses [195, 196]. Serglycin is highly expressed in the secretory granules of mast cells in connective tissues and the peritoneal cavity [197, 198]. Serglycin contains a large number of Ser-Gly repeats (8–24), to which HP or CS chains are attached [199, 200]. In connective tissue mast cells, serglycin is predominantly modified with HP chains. The abundance of highly sulfated HP underlies the metachromatic staining of mast cell granules with toluidine blue and explains why mucosal tissues rich in mast cells are commonly used as a source of unfractionated HP for pharmaceutical and laboratory use.

Serglycin plays fundamental roles in mast cell biology, as its deletion in mice leads to multiple abnormalities [201–203]. Serglycin is required for normal mast cell granule maturation, as normally granulated mast cells are absent from the peritoneum of serglycin-null mice [201]. Several mast cell-specific proteases, including MCP-4, MCP-5, MCP-6, and carboxypeptidase A, are absent from mast cells in the peritoneum and ear tissue of serglycin-null mice despite normal protease mRNA levels, indicating that serglycin is required for protease storage rather than expression. In addition, other highly basic mast cell proteases, such as chymases, tryptases, cathepsin G, and granzyme B, form tight complexes with serglycin [196]. Both HP and CS-E chains on serglycin mediate these functions, as mast cells lacking NDST-2 [204, 205] or GalNAc4S6OST [206] exhibit phenotypes similar to those of serglycin-deficient mast cells, and mast cells overexpressing heparanase contain reduced amounts of mast cell-specific proteases [207].

In general, serglycin binding positively regulates mast cell protease activity. For instance, HP enhances chymase activity by serving as a platform that brings chymases and their substrates into closer proximity or by shielding chymases from inhibitory serpins [208, 209]. HP binding also increases tryptase stability, thereby promoting enzymatic activity [210]. In addition, serglycin can retain secreted enzymes near the mast cell surface or within the surrounding ECM. Together, these observations indicate that serglycin regulates mast cell protease storage, secretion, activity, and stability through post-translational mechanisms. However, serglycin was dispensable for normal secretion and activity of mast cell proteases in response to peritoneal *Toxoplasma gondii* infection [211], suggesting that certain pathogens may induce the expression and secretion of mast cell proteases via serglycin-independent mechanisms.

Serglycin also affects the cell biology of mast cells. Serglycin coordinates mast cell apoptosis with MCP-6 [212, 213], and in its absence, mast cells predominantly undergo necrosis. Activated mast cells from Wt mice can efficiently reduce levels of exogenously administered IL-6, IL-13, and IL-17, whereas serglycin-null mast cells cannot [214, 215]. In this setting, serglycin-dependent proteases likely mediate proteolytic degradation of these cytokines, suggesting that serglycin is an important negative regulator of cytokine-driven inflammatory responses. Together, these findings indicate that serglycin and the proteases it regulates are potential therapeutic targets in mast cell-mediated diseases, including a wide range of inflammatory disorders [216–218]. Interestingly, inhaled HP has long been reported to have a protective effect in asthma in humans [219–222]. Although the mechanism remains unclear, one possibility is that inhaled HP disrupts serglycin–protease complexes and inhibits proteases that promote asthma pathogenesis. Alternatively, it may attenuate Th2-cell responses by interfering with Th2 chemokines, as observed with SDC1 ectodomains in experimental allergic lung inflammation [117].

Interestingly, dense-core granules are specifically absent in CD8+ cytotoxic T cells from serglycin knockout mice [223]. Granzyme B and perforin are reduced in intracellular compartments of serglycin-deficient cytotoxic T cells, whereas their levels are increased in culture supernatants [223]. These findings suggest that serglycin controls dense-core granule maturation, intracellular storage, and secretion of granzyme B and perforin. Consistent with this role, the cytotoxic activity of serglycin-deficient T cells is reduced [223]. Other T cell effector molecules may also be regulated by serglycin, as an earlier study showed that chemokines are released from cytotoxic T cell intracellular granules in complex with HSPGs [224]. Additionally, elastase is absent in the granules of mature neutrophils in serglycin knockout mice, whereas other granule proteins are not affected, suggesting that serglycin selectively regulates elastase localization in neutrophils. Like in neutrophil elastase-deficient mice, serglycin knockout mice are hypersusceptible to intraperitoneal infection with *K. pneumoniae* [225]. Overall, these results indicate that serglycin can influence granule biogenesis and protease activity in multiple hematopoietic cell types. However, because intense toluidine blue metachromasia is most prominent in mast cell granules, the intracellular abundance of serglycin is likely much lower in other leukocytes.

## 8 | Concluding Remarks

We now have a broader understanding of how HSPGs influence immunity by regulating signaling, adhesion, proliferation, differentiation, migration, and death of immune cells, as well as their ability to combat both endogenous and exogenous threats.

However, further advancement of the field requires a deeper understanding of the precise mechanisms and molecular specificity involved. For example, the chemokine gradient paradigm is an elegant concept, yet it remains oversimplified and lacks direct experimental validation in several key areas. The presumed role of HSPGs in this paradigm often assumes comparable levels of expression, similar chemokine-binding avidity, and equivalent effects on chemokine activity across different tissue compartments. These assumptions are unlikely to hold because distinct HSPGs bind and regulate chemokines with variable affinities. Moreover, the identity of the specific HSPGs that interact with chemokines remains largely unknown. Many studies have examined HS or HP function in bulk, leaving the cellular sources and identities of immunoregulatory HSPGs unresolved in most contexts. In addition, HSPG structure and function are dynamically regulated during inflammation, both during biosynthesis and after synthesis by heparanases, sulfatases, and sheddases, which can profoundly alter their biological activity. These layers of regulation are likely to influence the formation and stability of chemokine gradients, yet these aspects have not been thoroughly investigated.

Future research could also address several outstanding questions. Are the functions of HSPGs in immunity conserved across evolution? For example, do Drosophila SDC, Dally, and Dally-like protein regulate antimicrobial peptides and hemocyte activities in ways analogous to the roles of SDC1 and GPC4 in mammalian innate immunity? Does the appearance of SDCs and GPCs involved in adaptive immunity coincide with the emergence of specific cytokines, immune cell types, or signaling pathways associated with adaptive immune responses? What drives the distinct expression of SDC4 in mature B cells and SDC1 in plasma cells, and what underlies their presumably nonredundant functions in B cell development and function? Furthermore, what functions do HSPGs serve in rare immune subsets such as NKT17 and Tγδ17 cells?

Progress in studying the role of HSPGs in immunity will also require standardized experimental tools and criteria for assessing HSPG structure and activity. For example, many studies have not rigorously verified that the molecules under examination are true proteoglycans; native HSPGs should appear as diffuse smears rather than discrete protein bands on Western blots. The use of validated antibodies is essential for accurate detection. Investigators should also recognize that HSPG expression patterns in cultured cells may not reflect their in vivo profiles. For instance, endothelial cells, which typically do not express SDC1 in vivo, often upregulate SDC1 expression in vitro as part of tissue culture adaptation. Finally, the roles of HSPGs in pathological immune contexts remain poorly understood. Expanding research in this area may uncover novel druggable targets and further illuminate the molecular specificity and biological functions of HSPGs in immunity.

## Data Availability

The data that support the findings of this study are available from the corresponding author upon reasonable request.
